# Low Back Pain and Psychosocial Work Factors Associated with Sleep Problems among University and Hospital Employees: A Cross-sectional Study

**DOI:** 10.31662/jmaj.2026-0211

**Published:** 2026-08-28

**Authors:** Tomoko Tetsunaga, Takashi Hirai, Nobuyuki Fujita, Hiroaki Nakashima, Masakazu Minetama, Takaya Narita, Yuri Tamura, Tomohisa Nitta, Takuya Nikaido, Noriaki Kurita, Toshitaka Yoshii, Nobuhide Hirai, Mikako Kubo, Misaki Hattaha, Ryushi Tazawa, Seiji Ohtori

**Affiliations:** 1 Faculty of Human Life Sciences, Notre Dame Seishin University, Okayama, Japan; 2 Department of Sports Medicine, Faculty of Medicine, Dentistry and Pharmaceutical Sciences, Okayama University, Okayama, Japan; 3 Department of Orthopedic Surgery, Institute of Science Tokyo, Tokyo, Japan; 4 Department of Orthopedic Surgery, School of Medicine, Fujita Health University, Toyoake, Japan; 5 Department of Orthopedic Surgery, Nagoya University Graduate School of Medicine, Nagoya, Japan; 6 Department of Rehabilitation, Wakayama Medical University Kihoku Hospital, Katsuragi-cho, Ito-gun, Wakayama, Japan; 7 Department of Sport Technology, Faculty of Culture and Sport Policy, Toin University of Yokohama, Yokohama, Japan; 8 Department of Orthopaedic Surgery, Fukushima Medical University School of Medicine, Fukushima, Japan; 9 Department of Orthopaedic Surgery, Southern TOHOKU Research Institute for Neuroscience, Soutnern TOHOKU General Hospital, Kohriyama, Japan; 10 Department of Preventive Medicine, Tokai University School of Medicine, Isehara, Japan; 11 Student Support Center, Institute of Science Tokyo, Tokyo, Japan; 12 Faculty and Staff Healthcare Center, Institute of Science Tokyo, Tokyo, Japan; 13 Student Healthcare Center, Institute of Science Tokyo, Tokyo, Japan; 14 Department of Orthopaedic Surgery, Graduate School of Medicine, Chiba University, Chiba, Japan

**Keywords:** sleep problems, low back pain, healthcare workers, psychosocial work factors, occupational health

## Abstract

**Introduction:**

Sleep problems are common among healthcare workers, but associated factors among employees at a medical university and its affiliated hospital remain incompletely understood. This study examined psychosocial and clinical factors associated with sleep problems, with particular attention to low back pain (LBP).

**Methods:**

We conducted a cross-sectional, web-based survey of 2,450 employees from a medical university and its affiliated hospital using Brief Job Stress Questionnaire data from Japan’s legally mandated stress-check program in 2022. Sleep problems and LBP were assessed using single items. Age group, job category, body mass index, psychosocial work factors, other physical and psychological symptoms, and job and life satisfaction were also obtained. Group comparisons were performed between participants with and without sleep problems, and multivariable logistic regression was used to estimate odds ratios (ORs) and 95% confidence intervals (CIs).

**Results:**

Of 2,450 participants, 472 (19.3%) had sleep problems. In multivariable analyses adjusted for job category, LBP remained independently associated with higher odds of sleep problems (adjusted OR 1.16, 95% CI 1.03-1.30). Poor gastrointestinal condition, constipation or diarrhea, interpersonal stress at work, fatigue, and depressive mood were also independently associated with higher odds of sleep problems, whereas job and life satisfaction was associated with lower odds. In the dose–response analysis, the adjusted ORs for sleep problems increased across LBP categories after adjustment for job category and other covariates, with a significant linear trend (p for trend = 0.014).

**Conclusions:**

Among employees at a medical university and its affiliated hospital, LBP showed an independent and dose–response association with sleep problems after accounting for psychosocial work factors, clinical stress-related symptoms, and job category. By simultaneously evaluating musculoskeletal symptoms and psychosocial occupational factors in a large multiprofessional workforce, this study supports integrated occupational health approaches targeting both LBP and modifiable workplace factors.

## Introduction

Sleep problems are highly prevalent among healthcare workers and represent a major occupational health concern. Recent systematic reviews and meta-analyses report that approximately one-third to nearly half of healthcare workers experience insomnia symptoms or poor sleep quality, with prevalence estimates generally exceeding those in the general population ^(1)^, reflecting the impact of high job demands, irregular working hours, and emotionally demanding clinical environments. Poor sleep in this group has been linked to fatigue, impaired functioning, and reduced quality of care ^(2), (3)^.

Work-related stress is widely regarded as a major contributor to sleep problems in healthcare workers. Studies in various professional groups have shown that higher levels of general and occupational stress, work strain, and adverse psychosocial work environments are associated with insomnia symptoms and poor sleep quality ^(4), (5), (6), (7), (8)^. Low back pain (LBP) is also one of the most common occupational health problems among healthcare workers, reflecting frequent patient handling, prolonged standing, and physically demanding tasks. In parallel, research in both general and clinical populations indicates a close relationship between sleep problems and musculoskeletal symptoms, particularly chronic LBP, with accumulating evidence that sleep problems and chronic pain influence each other over time ^(9), (10), (11)^.

However, these two lines of research—work-related stress and sleep problems on the one hand and LBP and sleep problems on the other—have largely been examined separately. Only a limited number of studies have simultaneously evaluated psychosocial work factors and LBP as correlates of sleep problems within the same healthcare workforce, and the extent to which LBP is associated with sleep problems independent of key psychosocial characteristics remains largely unclear. Using large-scale Brief Job Stress Questionnaire (BJSQ) data collected through Japan’s legally mandated stress-check program from university faculty and staff as well as hospital employees, we were able to assess a broad range of psychosocial work factors and clinical symptoms across a large, multiprofessional workforce. Therefore, this cross-sectional study aimed to examine the association of sleep problems with both work-related psychosocial factors and LBP in a large, multiprofessional sample of employees from a medical university and its affiliated hospital. Our goal was to identify clinical and organizational targets for improving sleep health in this population.

## Materials and Methods

### Ethics statement

Ethics approval for this study was obtained from the ethics committee of the Institute of Science Tokyo, the coordinating institution (approval number: I2024-103). The study was conducted in accordance with relevant guidelines and regulations. The requirement for written informed consent was waived, and an opt-out approach was used by providing study information on institutional posters and websites. In accordance with the instruction of the ethics committee, sex information was not included in the anonymized research dataset provided to the investigators in order to minimize the risk of reidentification of participants in job categories with highly skewed sex distributions.

### Study design and participants

This study was a cross-sectional, web-based survey of employees affiliated with the Institute of Science Tokyo, including university faculty and staff and employees of its affiliated hospital. Eligible participants were employees affiliated with the Institute of Science Tokyo who completed both the stress-check survey and the annual health checkup in 2022. We excluded individuals who declined participation via the opt-out procedure; were younger than 20 years or older than 70 years; were not employed as healthcare professionals, researchers, faculty members, or administrative staff; or had incomplete survey or health checkup data. Demographic and occupational characteristics, including age group and job category, were obtained from the web-based survey, whereas body mass index (BMI) was measured during the annual health checkup and categorized according to World Health Organization criteria as underweight (<18.5 kg/m^2^), normal weight (18.5-24.9 kg/m^2^), overweight (25.0-29.9 kg/m^2^), and obese (≥30.0 kg/m^2^). Sex information was intentionally excluded from the anonymized research dataset provided to the investigators, following the instruction of the ethics committee, because including sex together with job category and other demographic variables could increase the risk of reidentification in occupational groups with highly skewed sex distributions.

In this sample, nurses represented the largest occupational group, followed by administrative/research staff, with co-medical professionals, physicians, and dentists comprising the remainder (Table 1). The cohort was predominantly young to middle-aged, with most participants in their 30s and 40s and relatively few aged 60 years or older. The mean BMI was 22.3 (standard deviation [SD] 3.6) kg/m^2^, and the majority of participants had a BMI within the normal range according to World Health Organization criteria, with smaller proportions classified as underweight, overweight, or obese.

**Table 1.** Participant Characteristics (n = 2,450).

Characteristics	Category	Participants, n (%)
Age group (years)	20s	571 (23.3)
	30s	728 (29.7)
	40s	632 (25.8)
	50s	404 (16.5)
	60s	115 (4.7)
BMI category (kg/m^2^)	Underweight (<18.5)	248 (10.1)
	Normal (18.5-24.9)	1,717 (70.1)
	Overweight (25.0-29.9)	391 (16.0)
	Obese (≥30)	94 ((3.8)
Job category	Nurse	661 (27.0)
	Administrative staff	565 (23.1)
	Research staff	540 (22.0)
	Co-medical staff	356 (14.5)
	Physician	235 (9.6)
	Dentist	93 (3.8)

BMI: body mass index.

The BJSQ is a 57-item instrument developed for the Japanese national stress-check program that assesses job stressors, psychological and physical stress responses, social support, and job and life satisfaction and has demonstrated good reliability and validity in occupational settings. Each item is rated on a four-point Likert scale ^(1), (2), (3), (4)^. Domain scores were calculated from the relevant BJSQ items according to the instrument manual. For items and subscales representing adverse states (e.g., mental and physical health symptoms, job stressors, and stress reactions), higher scores reflect more severe symptoms or more adverse psychosocial conditions, whereas higher scores on job control, supervisor support, family/friend support, and satisfaction scales reflect more favorable conditions (i.e., better control, support, or satisfaction). The primary outcome was the presence of sleep problems, assessed using the BJSQ item “I haven’t been able to sleep well” referring to the preceding month, rated on a four-point Likert scale (1 = almost never, 2 = sometimes, 3 = often, and 4 = almost always). Participants who responded 1 or 2 were classified into the no sleep problems group, and those who responded 3 or 4 were classified into the sleep problems group. Univariable analyses were then conducted to compare demographic, occupational, and clinical/psychosocial variables between these two groups.

### Statistical analysis

Continuous variables were summarized as mean (SD) and categorical variables as numbers and percentages. Baseline characteristics were compared between participants with and without sleep problems using Student’s t test for continuous variables and the chi-square test for categorical variables, as appropriate. All statistical tests were two-sided, and p < 0.05 was considered statistically significant. Statistical analyses were performed using SPSS software version 25.0 for Windows (IBM Corp., Armonk, NY, USA).

To examine factors associated with sleep problems, we first constructed an exploratory multivariable logistic regression model with the presence of sleep problems (yes/no) as the dependent variable. Effect sizes (ESs) for continuous variables were expressed as standardized mean differences (Cohen’s d), calculated as the difference in group means divided by the pooled SD, whereas effect sizes for categorical variables were expressed as Cramér’s V, calculated from the chi-square statistic. Following commonly used guidelines, Cohen’s d values of approximately 0.2, 0.5, and 0.8 and Cramér’s V values of approximately 0.1, 0.3, and 0.5 were interpreted as small, moderate, and large effects, respectively. Because this first multivariable model was intended as an exploratory analysis to screen correlates of sleep problems from a relatively large set of candidate variables, candidate continuous-scale variables with Cohen’s d ≥ 0.50 in univariable analyses were entered into the model in order to limit the number of predictors and reduce the risk of overfitting. Odds ratios (ORs) with 95% confidence intervals (CIs) were calculated.

Second, to specifically examine the dose–response association between LBP and sleep problems, we fitted a separate multivariable logistic regression model with sleep problems (yes/no) as the dependent variable and LBP category as the main independent variable. In this second model, covariates were selected a priori based on previous studies linking gastrointestinal, psychological, and interpersonal stress-related factors to sleep problems and included poor gastrointestinal condition, bowel problems, interpersonal stress at work, fatigue, depressive mood, and job and life satisfaction ^(12), (13), (14), (15), (16)^. LBP was assessed using the item “low back pain” with four response categories (1 = almost never, 2, 3, and 4 = almost always). To evaluate a possible dose–response relationship, LBP was entered into the model as a categorical variable, treating category 1 as the reference group and estimating ORs and 95% CIs for categories 2-4. In addition, to formally test for a linear trend across LBP categories, we entered the four-level LBP variable as a single ordinal term ^(1), (2), (3), (4)^ and used the Wald test for its regression coefficient; the resulting p-value was reported as the p for trend. Sex information was intentionally not included in the anonymized research dataset provided to the investigators, in accordance with instruction of the ethics committee, to minimize the risk of reidentification of participants in job categories with highly skewed sex distributions. Therefore, sex could not be included as a covariate in the multivariable models. Job category was included as an occupational covariate in the multivariable logistic regression models. Job category was treated as a categorical variable, with administrative staff used as the reference group. All variables entered into the main multivariable model are presented in Table 4, regardless of statistical significance. For the dose–response analysis, all adjustment variables are described in the footnote to Table 5.

## Results

Among the 3,856 individuals eligible for the stress check, 2,883 (74.8%) completed the web-based survey. Of these, 2,450 participants met the eligibility criteria and had complete survey and health checkup data and were included in the analysis. Among these 2,450 employees from a medical university and its affiliated hospital, 1,978 (80.7%) were classified as having no sleep problems and 472 (19.3%) as having sleep problems based on the stress-check item “I haven’t been able to sleep well.” Age (p = 0.56) and BMI distributions (p = 0.73) were broadly similar between the two groups, and their ESs were small; therefore, these variables were not included in the multivariable model. Baseline characteristics of the two groups are summarized in Table 2.

**Table 2.** Participant Characteristics (n = 2,450).

Characteristics	Category	No sleep problems (n = 1,978), n (%)	Sleep problems (n = 472), n (%)	p-Value	ES
Age group (years)	20s	456 (23.1)	115 (24.4)	0.56^a^	0.035^b^
	30s	597 (30.2)	131 (27.8)		
	40s	510 (25.8)	122 (25.8)		
	50s	328 (16.6)	76 (16.1)		
	60s	87 (4.4)	28 (5.9)		
BMI (kg/m^2^), mean (SD)		22.36 (3.63)	22.22 (3.75)	0.45^c^	0.039^d^
BMI category	Underweight (<18.5)	195 (9.9)	53 (11.2)	0.73^a^	0.023^b^
	Normal (18.5-24.9)	1,389 (70.2)	328 (69.5)		
	Overweight (25.0-29.9)	320 (16.2)	71 (15.0)		
	Obese (≥30)	74 (3.7)	20 (4.2)		
Job category	Nurse	491 (24.8)	170 (36.0)	<0.001^a^	0.13^b^
	Administrative staff	449 (22.7)	116 (24.6)		
	Research staff	451 (22.8)	89 (18.9)		
	Co-medical staff	292 (14.8)	64 (13.6)		
	Physician	215 (10.9)	20 (4.2)		
	Dentist	80 (4.0)	13 (2.8)		

Note: Differences were considered significant at p < 0.05. ^a^chi-square test. ^b^Cramér’s V. ^c^Student’s t test. ^d^Cohen’s d. BMI: body mass index; ES: effect size; SD: standard deviation.

In the univariate analyses, participants with sleep problems reported higher levels of depressive mood, fatigue, poor gastrointestinal condition, constipation or diarrhea, poor appetite, interpersonal stress at work, LBP, psychological job demands, and perceived physical burden compared with those without sleep problems (all p < 0.001, Table 3). In contrast, job and life satisfaction, job control, and supervisor as well as family and friend support were significantly lower in the sleep problems group (all p < 0.001). Large ESs were observed for depressive mood (d = 1.04), fatigue (d = 0.92), and poor gastrointestinal condition (d = 0.82). Moderate ESs were observed for constipation or diarrhea (d = 0.72), job and life satisfaction (d = 0.69), poor appetite (d = 0.68), interpersonal stress at work (d = 0.58), and LBP (d = 0.59).

**Table 3.** Univariate Analysis.

Variables	No sleep problems (n = 1,978), mean (SD)	Sleep problems (n = 472), mean (SD)	p-Value	ES
Depressive mood	2.55 (1.09)	3.68 (0.98)	<0.001	1.04
Fatigue	2.94 (0.93)	3.79 (0.89)	<0.001	0.92
Poor gastrointestinal condition	1.53 (0.75)	2.18 (0.99)	<0.001	0.82
Constipation or diarrhea	1.64 (0.83)	2.26 (0.99)	<0.001	0.72
Job and life satisfaction	3.39 (0.84)	2.81 (0.83)	<0.001	0.69
Poor appetite	1.29 (0.56)	1.71 (0.82)	<0.001	0.68
Low back pain	1.87 (0.95)	2.44 (1.06)	<0.001	0.59
Interpersonal stress at work	2.78 (0.90)	3.31 (0.96)	<0.001	0.58
Job control	3.36 (0.86)	2.93 (0.90)	<0.001	0.496
Supervisor support	3.39 (1.00)	2.93 (1.06)	<0.001	0.46
Family and friend support	3.65 (1.31)	3.11 (1.48)	<0.001	0.40
Psychological job demands (quantitative)	3.24 (0.90)	3.54 (0.93)	<0.001	0.34
Perceived physical burden	3.39 (0.96)	3.61 (1.06)	<0.001	0.22

Notes: Poor gastrointestinal condition, constipation or diarrhea, poor appetite, and low back pain were assessed as single items scored on a four-point scale (1-4); the remaining variables were Brief Job Stress Questionnaire domain scores calculated from multiple items. Differences were considered statistically significant at p < 0.05. p- Values were calculated using Student’s t test, and ESs are presented as Cohen’s d. ES: effect size; SD: standard deviation.

In the multivariable logistic regression analysis, several clinical and psychosocial factors were independently associated with sleep problems (Table 4). LBP remained independently associated with higher odds of sleep problems (adjusted OR 1.16, 95% CI 1.03-1.30; p = 0.017). Poor gastrointestinal condition, constipation or diarrhea, interpersonal stress at work, fatigue, and depressive mood were also associated with increased odds of sleep problems, whereas higher job and life satisfaction was associated with lower odds of sleep problems. Job category was included in the model as an occupational covariate and is presented in Table 4 together with all other variables entered into the model, regardless of statistical significance. In the revised multivariable model, nurses did not have significantly higher odds of sleep problems than administrative staff (adjusted OR 1.09, 95% CI 0.80-1.49; p = 0.580). All variance inflation factors were below 2.2, indicating no problematic multicollinearity among covariates (Supplementary Table S1).

**Table 4.** Multivariable Logistic Regression Analysis of Factors Associated with Sleep Problems.

Variables	OR (95% CI)	p-Value
Poor appetite	1.16 (0.97-1.39)	0.096
Constipation or diarrhea	1.36 (1.18-1.56)	<0.001
Interpersonal stress at work	1.21 (1.05-1.39)	0.007
Fatigue	1.50 (1.27-1.77)	<0.001
Depressive mood	1.60 (1.38-1.85)	<0.001
Job and life satisfaction	0.74 (0.63-0.88)	<0.001
Job category		
Administrative staff	1.00 (ref)	
Nurse	1.09 (0.80-1.49)	0.580
Research staff	1.09 (0.76-1.54)	0.645
Co-medical staff	0.74 (0.50-1.09)	0.124
Physician	0.60 (0.34-1.04)	0.069
Dentist	0.45 (0.22-0.92)	0.028
Constant term	0.005 (0.002-0.013)	<0.001

Notes: The model included low back pain, poor gastrointestinal condition, poor appetite, constipation or diarrhea, interpersonal stress at work, fatigue, depressive mood, job and life satisfaction, and job category. Job category was treated as a categorical variable, with administrative staff as the reference group. Differences were considered statistically significant at p < 0.05. CI: confidence interval; OR: odds ratio; ref: reference.

The prevalence of sleep problems increased monotonically across LBP categories. Among participants with the lowest level of LBP (category 1), 11.2% reported sleep problems, compared with 18.0%, 28.1%, and 39.4% in categories 2, 3, and 4, respectively (Table 5). In multivariable logistic regression analyses adjusting for job category, poor gastrointestinal condition, bowel problems, interpersonal stress at work, fatigue, depressive mood, and job and life satisfaction, the adjusted ORs for sleep problems increased across LBP categories. Using category 1 as the reference, the adjusted ORs (95% CIs) for sleep problems were 1.15 (0.85-1.55) for category 2, 1.34 (0.97-1.85) for category 3, and 1.56 (1.06-2.30) for category 4. When LBP was treated as an ordinal variable ^(1), (2), (3), (4)^, each one-category increase in LBP was associated with a 16% higher odds of sleep problems (adjusted OR 1.16, 95% CI 1.03-1.31), and the linear trend across categories was statistically significant (p for trend = 0.014), indicating a dose–response relationship after adjustment for job category and other covariates.

**Table 5.** Association between Categories of Low Back Pain and Sleep Problems (Multivariable-Adjusted).

Category of low back pain	n	Cases of sleep problems, n (%)	Adjusted OR^a^ (95% CI)	p-Value
1 (lowest)	998	112 (11.2)	1.00 (ref)	NA
2	739	133 (18.0)	1.15 (0.85-1.55)	0.366
3	477	134 (28.1)	1.34 (0.97-1.85)	0.077
4 (highest)	236	93 (39.4)	1.56 (1.06-2.30)	0.023

Notes: Job category was treated as a categorical variable, with administrative staff as the reference group. When low back pain was treated as an ordinal variable, p for trend = 0.014. Differences were considered statistically significant at p < 0.05. ^a^Adjusted for job category, poor gastrointestinal condition, bowel problems, interpersonal stress at work, fatigue, depressive mood, and job and life satisfaction. CI: confidence interval; NA: not applicable; OR: odds ratio; ref: reference.

## Discussion

In this large, multiprofessional sample of 2,450 university faculty and staff as well as hospital employees, approximately one in five workers had sleep problems. Sleep problems clustered with several adverse psychosocial and clinical conditions, such as higher levels of depressive mood, fatigue, poor gastrointestinal condition, interpersonal stress at work, and LBP, whereas higher job and life satisfaction were independently associated with a lower likelihood of sleep problems. Moreover, the prevalence and adjusted odds of sleep problems increased monotonically across LBP categories, and treating LBP as an ordinal variable revealed a significant linear trend, indicating a dose–response relationship after adjustment for job category and key psychosocial/clinical covariates. Taken together, these findings suggest that both adverse psychosocial work factors and LBP are independently associated with sleep problems among healthcare workers, highlighting multiple clinical and organizational targets for interventions to improve sleep health in this population.

A unique contribution of this study is that it simultaneously assessed LBP, psychosocial work factors, and clinical stress-related symptoms in a large multiprofessional sample of university and hospital employees using data from Japan’s legally mandated stress-check program. Previous studies have often examined occupational stress and sleep problems or LBP and sleep disturbance separately. In contrast, this study evaluated these factors within the same workforce and showed that LBP was independently associated with sleep problems in a dose-dependent manner, even after adjustment for psychosocial/clinical factors and job category.

Although nurses were more common among participants with sleep problems in the univariable comparison, nursing occupation was not significantly associated with sleep problems in the revised multivariable model using administrative staff as the reference category. This finding suggests that the higher crude prevalence of sleep problems among nurses may have been partly explained by clinical and psychosocial factors included in the model. However, because sex information was intentionally not included in the anonymized research dataset in accordance with the ethics committee’s instruction to protect participant anonymity, residual confounding by sex cannot be excluded, particularly in the interpretation of findings related to nursing occupation.

Our observation that LBP is clearly linked to sleep problems is consistent with a large body of evidence demonstrating a close relationship between sleep problems and chronic musculoskeletal pain. Prospective cohort studies in general populations have shown that insomnia symptoms and short sleep duration increase the risk of developing chronic spinal or multisite musculoskeletal pain by roughly 30%-70%, establishing sleep problems as a predictor of incident pain rather than merely a consequence ^(17), (18), (19)^. Mendelian randomization studies further support a causal effect of insomnia on LBP, with genetically predicted insomnia conferring higher odds of LBP ^(20), (21)^. Systematic reviews and meta-analyses also indicate bidirectional associations: sleep problems at baseline increase the incidence and persistence of chronic musculoskeletal pain, and chronic pain, in turn, increases the risk of subsequent sleep problems ^(10), (11)^. Together with reports that healthcare workers frequently experience both musculoskeletal complaints and sleep problems ^(4)^, these findings reinforce the clinical significance of the association we observed between LBP and sleep problems among employees of a medical university and its affiliated hospital. LBP may serve as both a marker and a potential contributor to sleep problems in high-demand occupations.

At the same time, some studies suggest a more complex or even asymmetrical relationship between sleep and LBP, which tempers causal interpretation of our cross-sectional findings. Agmon and Armon reported in a cohort of employed adults that increases in insomnia symptoms predicted new-onset back pain, whereas back pain did not predict subsequent insomnia, implying a predominantly sleep-to-pain direction in that population ^(19)^. Similarly, a Mendelian randomization study found strong evidence that genetically predicted insomnia increases the risk of LBP but no evidence that genetically predicted LBP increases insomnia or daytime sleepiness ^(21)^. These data do not contradict the association we observed, but they caution against assuming a simple causal pathway from LBP to sleep problems and suggest that shared psychological or lifestyle factors or a preexisting vulnerability to insomnia may partly account for our findings. Nonetheless, by demonstrating a robust association between LBP and sleep problems after adjustment for key work-related and clinical factors, our study underscores the importance of routinely assessing sleep problems in healthcare workers who report LBP.

The independent associations of interpersonal stress at work, fatigue, and depressive mood with sleep problems are in line with extensive literature on work-related stress and insomnia among healthcare workers. Cross-sectional and longitudinal studies have consistently shown that high job demands, work strain, and effort–reward imbalance are strongly linked to insomnia symptoms in nurses and other healthcare workers, often with approximately twofold higher odds of insomnia in high-stress groups compared with low-stress groups ^(2), (4), (5), (6), (7)^. Hämmig reported that general and work-related stress were significantly associated with both musculoskeletal complaints and sleep problems among hospital staff ^(4)^, while Çaltekin and Hamamcı showed that emergency department workers have higher work strain and poorer sleep quality than other healthcare workers ^(5)^. The prospective study by Linton suggested that eliminating job strain could prevent a substantial proportion of new insomnia cases, highlighting the potential impact of improving work conditions on sleep ^(7)^. More recently, structural equation modeling in nurses during the COVID-19 pandemic demonstrated that occupational stress exerts a direct effect on insomnia, partly mediated by resilience, and that resilience functions as a protective factor ^(8)^. In our sample, approximately 19% of workers were classified as having sleep problems, which is lower than the 40%-45% prevalence reported in meta-analyses and longitudinal studies during the COVID-19 pandemic ^(3), (6), (22)^. This discrepancy may reflect differences in measurement, time period, and workplace context.

This study has several limitations that should be considered when interpreting the findings. First, its cross-sectional design precludes any firm conclusions about causal or temporal relationships between sleep problems, LBP, and psychosocial work factors. Second, the data were obtained from a single medical university and its affiliated hospital through a web-based, voluntary stress-check survey, which may limit external validity and introduce selection bias; workers in other types of healthcare facilities or regions may have different characteristics. Because the survey was conducted within the framework of Japan’s legally mandated stress-check program, some participants may also have underreported symptoms or stress due to concerns—however unfounded—about potential occupational consequences. Third, sex information was intentionally not included in the anonymized research dataset provided to the investigators, in accordance with the instruction of the ethics committee, to minimize the risk of reidentification of participants in job categories with highly skewed sex distributions. This is an important limitation because sex is a fundamental demographic variable in studies of sleep problems, and the nursing workforce in Japan is predominantly female. Therefore, residual confounding by sex may have influenced the observed associations among job category, LBP, psychosocial work factors, and sleep problems. In particular, findings related to nursing occupation should be interpreted with caution because they may partly reflect unmeasured differences in sex distribution. Future studies should include sex-stratified and sex-adjusted analyses when such analyses can be conducted while adequately protecting participant anonymity. Finally, both sleep problems and LBP were assessed using single self-reported items from the BJSQ, without objective sleep measures or validated multidimensional pain instruments. As a result, misclassification is possible, and we could not distinguish specific sleep disorders or characterize the chronicity, etiology, and functional impact of LBP.

Despite these limitations, our findings indicate that occupational health approaches aimed at supporting sleep in healthcare workers may need to address both musculoskeletal symptoms—particularly LBP—and modifiable psychosocial aspects of the workplace, such as interpersonal relationships and job satisfaction. Future longitudinal and interventional studies in more diverse healthcare settings are warranted to clarify temporal relationships, explore underlying mechanisms, and evaluate whether improvements in pain management and the psychosocial work environment can contribute to better sleep and overall well-being among healthcare workers.

### Conclusion

In this cross-sectional study of employees from a medical university and its affiliated hospital, LBP was independently and dose-dependently associated with sleep problems after adjustment for psychosocial work factors, clinical stress-related symptoms, and job category. By simultaneously evaluating musculoskeletal symptoms and psychosocial occupational factors in a large multiprofessional workforce, this study suggests that occupational health strategies for sleep problems in university and hospital employees should consider both LBP and modifiable psychosocial aspects of the workplace.

## Article Information

### Acknowledgments

We would like to thank Editage (www.editage.com) for English language editing.

### Author Contributions

Conceptualization: Tomoko Tetsunaga, Takashi Hirai, Noriaki Kurita, Hiroaki Nakashima, Masakazu Minetama, Takaya Narita, Mikako Kubo, Ryushi Tazawa, Nobuyuki Fujita, and Seiji Ohtori. Formal analysis: Tomoko Tetsunaga, Takashi Hirai, Tomohisa Nitta, Noriaki Kurita, Nobuhide Hirai, Mikako Kubo, Misaki Hattaha, and Ryushi Tazawa. Funding acquisition: Takuya Nikaido. Investigation: Tomoko Tetsunaga, Takashi Hirai, Yuri Tamura, Tomohisa Nitta, Nobuhide Hirai, Mikako Kubo, Misaki Hattaha, and Ryushi Tazawa. Methodology: Tomoko Tetsunaga, Takashi Hirai, Nobuyuki Fujita, Noriaki Kurita, Nobuhide Hirai, Mikako Kubo, Misaki Hattaha, and Ryushi Tazawa. Supervision: Noriaki Kurita, Takuya Nikaido, Nobuyuki Fujita, Nobuhide Hirai, Misaki Hattaha, Ryushi Tazawa, Toshitaka Yoshii, and Seiji Ohtori. Writing – original draft: Tomoko Tetsunaga and Takashi Hirai. Writing – review and editing: Tomoko Tetsunaga, Takashi Hirai, Nobuyuki Fujita, Hiroaki Nakashima, Masakazu Minetama, Takuya Nikaido, Takaya Narita, Yuri Tamura, Tomohisa Nitta, Noriaki Kurita, Nobuhide Hirai, Mikako Kubo, Misaki Hattaha, Ryushi Tazawa, Toshitaka Yoshii, and Seiji Ohtori.

### Conflicts of Interest

None

### Ethics Approval

This study was approved by the ethics committee of the Institute of Science Tokyo (approval number: I2024-103).

## Supplement

Supplementary Material
