# Supplementary material for: Low Back Pain and Psychosocial Work Factors Associated with Sleep Problems among University and Hospital Employees: A Cross-sectional Study
Source: JMA J. 2026 Aug 28;9(5):1133–41. doi: 10.31662/jmaj.2026-0211 (PMC13639655; doi:10.31662/jmaj.2026-0211)
Supplement: Supplementary file 1 [file 2433-3298-9-5-1133-s001.pdf]

Supplemental Table 1 Variance inflation factors

Variables	R^2	VIF
Low back pain	0.178	1.22
Poor gastrointestinal condition	0.445	1.80
Poor appetite	0.254	1.34
Constipation or diarrhea	0.361	1.57
Interpersonal stress at work	0.220	1.28
Fatigue	0.452	1.83
Depressive mood	0.526	2.11
Job and life satisfaction	0.290	1.41
Job category: Nurse	0.390	1.64
Job category: Research staff	0.361	1.56
Job category: Co-medical staff	0.297	1.42
Job category: Physician	0.246	1.33
Job category: Dentist	0.118	1.13

VIF, variance inflation factor. R^2 values were obtained from auxiliary linear regression models in which each predictor was regressed on all other predictors. The model included low back pain, poor gastrointestinal condition, poor appetite, constipation or diarrhea, interpersonal stress at work, fatigue, depressive mood, job and life satisfaction, and job category. Job category was treated as a categorical variable, with administrative staff as the reference group.
